# No Dose Adjustment for Isavuconazole Based on Age or Sex

**DOI:** 10.1128/AAC.02629-18

**Published:** 2019-05-23

**Authors:** Amit V. Desai, David Han, Donna L. Kowalski, Christopher Lademacher, Helene Pearlman, Takao Yamazaki

**Affiliations:** a Astellas Pharma Global Development Inc., Northbrook, Illinois, USA; b Parexel, Los Angeles, California, USA; c Astellas Pharma Inc., Tokyo, Japan

**Keywords:** age, isavuconazole, pharmacokinetics, sex

## Abstract

This phase 1, open-label, single-dose, parallel-group study evaluated the pharmacokinetics (PK) of isavuconazole after a single oral dose of the prodrug isavuconazonium sulfate in healthy nonelderly (age, 18 to 45 years) and elderly (age, ≥65 years) males and females. Overall, 48 subjects were enrolled in the study (*n *=* *12 each in groups of nonelderly males and females and elderly males and females). All subjects received a single oral dose of 372 mg of isavuconazonium sulfate (equivalent to 200 mg isavuconazole). PK samples were collected for analysis of isavuconazole plasma concentrations from the predose time point up to 336 h postdose. Data were analyzed using population pharmacokinetic (PPK) analysis. The resulting PPK model included two compartments with Weibull absorption function as well as interindividual variability with respect to clearance, intercompartment clearance, volumes of central and peripheral compartments, and two Weibull absorption parameters, RA and KAMAX. The PPK analysis showed that elderly females had the highest exposure versus males (ratio of total area under the time-concentration curve [AUC], 138; 90% confidence interval [CI], 118 to 161) and versus nonelderly females (ratio of AUC, 147; 90% CI, 123 to 176). Higher exposures in elderly females were not associated with significant toxicity or treatment-emergent adverse events, as measured in this study. No dose adjustments appear to be necessary based on either age group or sex even with an increase in exposure for elderly females. (This study has been registered at ClinicalTrials.gov under registration no. NCT01657890.)

## Abstract (precis)

This phase 1, open-label, single-dose, parallel-group study evaluated the pharmacokinetics (PK) of isavuconazole after a single oral dose of the prodrug isavuconazonium sulfate in healthy nonelderly (age, 18 to 45 years) and elderly (age, ≥65 years) males and females. Overall, 48 subjects were enrolled in the study (*n *=* *12 each in groups of nonelderly males and females and elderly males and females).

## TEXT

Pharmacotherapy can be a challenging aspect of the care of elderly patients, as drug distribution, metabolism, and renal elimination can all be affected by age group (1). There is an increased risk of drug-drug interactions and other adverse drug effects in the elderly compared with younger subjects, which can make the use of pharmacological interventions in the elderly more problematic (1). Invasive fungal disease (IFD) is a growing problem in the elderly because they are more likely to require transplantation or to receive immunosuppressive drugs or chemotherapy for cancer than younger adults (2). The elderly are less able to cope with IFD, and outcomes are frequently worse than in younger adults (2).

Triazole antifungal agents are frequently first-line agents for the prevention and treatment of IFD in older adults or in immunocompromised patients with cancer or those requiring transplantation (3). Isavuconazonium sulfate is a water-soluble prodrug of the active triazole isavuconazole and inhibits the sterol 14 alpha-demethylase, a microsomal P450 enzyme (P45014DM) that is essential for ergosterol biosynthesis in fungi (4, 5). Based on the results of phase 3 clinical trials (6, 7), isavuconazonium sulfate was approved by the U.S. Food and Drug Administration for the treatment of invasive aspergillosis (IA) and invasive mucormycosis in adults (8). It was also approved by the European Medicines Agency for the treatment of IA in adults and those with mucormycosis, for whom amphotericin B is not appropriate (9).

It is crucial for the optimal clinical use of isavuconazole to understand its pharmacokinetics (PK) in various patient populations (10–13), including nonelderly and elderly male and female adults. The primary objectives of this study were to determine the PK profiles of isavuconazole after a single dose in healthy nonelderly and elderly adults and in male and female adult subjects and to determine if dose adjustment is needed based on age group or sex.

## RESULTS

### Demographics and baseline characteristics.

A total of 48 healthy adults were enrolled in the study, including 12 female and 12 male nonelderly adults and 12 female and 12 male elderly adults (Table 1). The proportion of white subjects enrolled in the nonelderly group (13/24 [54.2%]) was similar to the proportion of those enrolled in the elderly group (11/24 [45.8%]). The nonelderly group had a higher proportion of black or African American subjects (7/24 [29.2%]) than the elderly group (1/24 [4.2%]). The mean weight and body mass index (BMI) data were comparable between the nonelderly and elderly groups (Table 1). Across the age groups, there were more white male subjects (17/24 [70.8%]) than white female subjects (7/24 [29.2%]). However, there were more black or African American (7/24 [29.2%]) and Asian (9/24 [37.5%]) female subjects across the age groups than black or African American and Asian male subjects (1/24 [4.2%] and 4/24 [16.7%], respectively). At least 25% of the elderly subjects (three from each sex) were above 75 years of age.

**TABLE 1:** Participant demographics

Parameter^*a*^	Value(s) for indicated patient group			
	Nonelderly		Elderly	
	Male (*n* = 12)	Female (*n* = 12)	Male (*n* = 12)	Female (*n* = 12)
Ethnicity, *n* (%)				
White	9 (75)	4 (33.3)	8 (66.7)	3 (25.0)
Black or African American	1 (8.3)	6 (50.0)	0	1 (8.3)
Asian	0	1 (8.3)	4 (33.3)	8 (66.7)
American Indian or Alaskan	1 (8.3)	0	0	0
Other	1 (8.3)	1 (8.3)	0	0
Age (yrs), mean ± SD	30.3 ± 7.59	29.5 ± 6.45	70.9 ± 5.74	71.5 ± 5.50
Range	19–42	22–45	65–85	66–84
Wt, mean kg ± SD	80.17 ± 12.90	70.01 ± 10.49	77.88 ± 11.58	61.03 ± 8.71
Range	57.0–105.7	56.3–86.5	59.1–94.1	51.7–80.0
BMI, mean kg/m^2^ ± SD	26.44 ± 3.32	26.17 ± 3.47	25.98 ± 2.36	24.52 ± 3.28
Range	20.2–31.7	19.6–30.9	22.5–29.8	19.7–30.0

a BMI, body mass index; SD, standard deviation.

### Population pharmacokinetic model.

**FIG 1:**
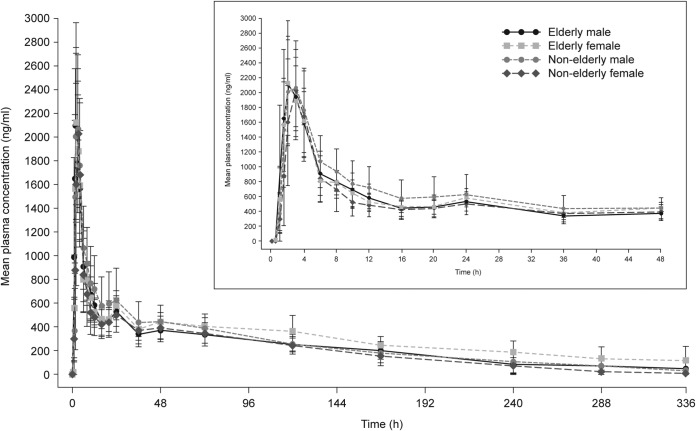
Means ± standard deviations of plasma concentrations of isavuconazole by age group and sex in the pharmacokinetic analysis set. The inset shows an expanded 0-to-48-h interval.

**FIG 2:**
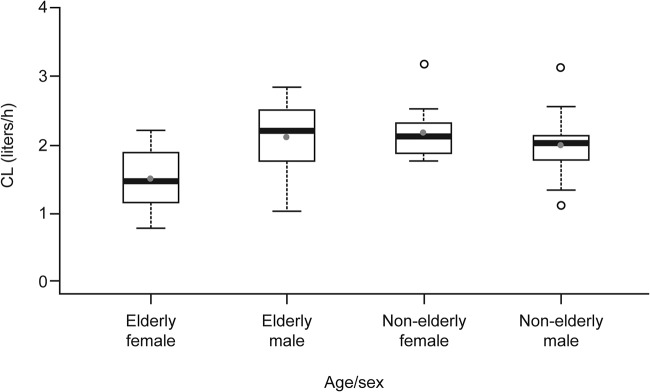
Isavuconazole clearance values for the different groups based on age and sex in subjects from the pharmacokinetic analysis set. Boxes represent medians and 25th and 75th percentiles, whiskers represent 1.5× the interquartile range, solid gray circles represent means, and open circles represent outliers. CL, clearance.

The plasma concentrations of isavuconazole showed a biexponential decline after the peak plasma concentration was achieved, with a prolonged terminal elimination phase (Fig. 1). The initial population PK (PPK) model resulted in a two-compartment model, with unique clearance (CL) values for each group (elderly male, nonelderly male, elderly female, and nonelderly female). The isavuconazole CL values were similar for the nonelderly male subjects (CL ± standard deviation [SD], 1.94 ± 0.52 liters/h) and elderly male subjects (2.04 ± 0.52 liters/h), whereas the CL values differed between the nonelderly and elderly females (2.13 ± 0.39 liters/h and 1.44 ± 0.43 liters/h, respectively). (The CL values are presented as box plots in Fig. 2.) Therefore, in the modified base model, nonelderly and elderly male subjects were evaluated as a single group whereas nonelderly and elderly female subjects were evaluated as separate distinct groups. The PK model development process resulted in a modified base model that included two compartments with Weibull absorption function. The model had interindividual variability with respect to CL, intercompartmental clearance (Q), volumes of central and peripheral compartments (*V*_2_ and *V*_3_, respectively), and two Weibull absorption parameters (RA and KAMAX). Modeling of isavuconazole PPK data then proceeded with an exploratory graphical inspection of potential covariate parameter relationships for the primary covariate of interest.

### Best model with covariates.

Following the development of the modified base model, covariates of interest were added in a stepwise manner using the forward-inclusion/backward-elimination procedure. The only covariate statistically significant on *V*_3_ was weight:

$$V_{3}=\theta_{4}\times \left[1+\theta_{9}\times \left(\text{WTKG}-72\right)\right]$$

where θ_4_ is the typical value of volume of distribution, θ_9_ is the value of volume of distribution associated with changing weight, and WTKG is weight in kilograms. The diagnostics of the model fit (see Fig. S1 in the supplemental material) indicated an overall good fit of the model to the data. Typical population parameters, including covariate effects as well as most of the random variance parameters, were estimated with good precision. Plots of the normalized prediction distribution error (NPDE; Fig. S2) demonstrated that the normality assumption was met, and plots of NPDE versus time (independent variable) did not show any trend. The parameters for the best covariate model are presented in Table 2. All parameters were precisely estimated with percent relative standard errors of ≤39% for the fixed effects and ≤40% for the random effects. Clearance shrinkage was 5%. The condition number of the two-compartment model was 65, indicating stability of the model.

**TABLE 2:** Parameter estimates of the best covariate model^*a*^

Parameter	Value	% RSE	Bootstrap mean	Bootstrap 95% CI
θ_1_ (CL, male), liters/h	1.99	6	1.98	1.75–2.23
θ_2_ (CL, nonelderly female), liters/h	2.13	6	2.13	1.87–2.38
θ_3_ (CL, elderly female), liters/h	1.44	10	1.45	1.17–1.71
θ_4_ (V2), ml	9,220	14	9,140	3,993–14,450
θ_5_ (Q), ml/h	22,200	4	22,190	20,252–24,092
θ_6_ (V3), ml	263,000	5	264,231	234,000–292,835
θ_7_ (RA)	0.664	6	0.661	0.574–0.735
θ_8_ (GAM1)	4.75	10	4.77	3.62–5.87
θ_9_ (KAMAX), h^−1^	0.426	6	0.42	0.368–0.482
θ_10_ V3 WTKG, Male	0.0212	10	0.021	0.0141–0.028
θ_11_ V3 WTKG, nonelderly female	0.443	39	0.458	0.1–0.786
θ_12_ V3 WTKG elderly female	0.654	23	0.663	0.204–1.10

a RSE, relative standard error; CI, confidence interval; CL, clearance; V2, volume of central peripheral compartment; V3, volume of peripheral compartment; Q, intercompartmental clearance; WTKG, body weight; GAM1, KAMAX, and RA, Weibull absorption parameters.

**FIG 3:**
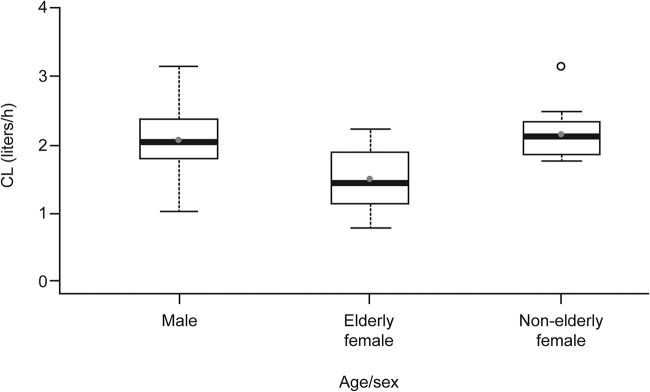
Isavuconazole clearance values from the two-compartment model in the pharmacokinetic analysis set. Boxes represent medians and 25th and 75th percentiles, whiskers represent 1.5× the interquartile range, solid gray circles represent means, and open circles represent outliers. CL, clearance.

Area under the concentration-time curve (AUC) data were calculated using empirical Bayes estimates. Similar isavuconazole CL values were evident for the male and nonelderly female subjects (Fig. 3). The elderly females had the highest exposure of the groups studied (ratio of elderly females to males, 138; 90% confidence interval [CI], 118 to 161; ratio of elderly females to nonelderly females, 147; 90% CI, 123 to 176). The AUC values determined for the nonelderly females were similar to the data determined for the combined male groups (ratio, 94; 90% CI, 80 to 109).

### Safety analyses.

Overall, 15 subjects (31.3%) reported 19 treatment-emergent adverse events (TEAEs) during the study (Table 3). Elderly females reported the highest number of TEAEs. Elderly females also reported the highest number of drug-related TEAEs (five TEAEs in four subjects [33.3%]) compared with elderly men (0), nonelderly females (two TEAEs in one subject [8.3%]), and nonelderly males (one TEAE in one subject [8.3%]). All TEAEs were mild in intensity. There were no serious adverse events and no deaths during the study. The most common MedDRA (Medical Dictionary for Regulatory Activities) system organ class TEAE was represented by gastrointestinal disorders occurring in two (16.7%) nonelderly males and three (25%) nonelderly females. There were no clinically meaningful changes from baseline for chemistry or hematology results. Overall, the mean vital sign measurements at baseline were similar to the mean vital sign measurements after dosing, with no clinically relevant changes. No subject experienced a clinically significant 12-lead electrocardiogram (ECG) abnormality during the study.

**TABLE 3:** Summary of treatment-emergent adverse events

System order class preferred term	No. (%) of patients			
	Nonelderly		Elderly	
	Male (*n* = 12)	Female (*n* = 12)	Male (*n* = 12)	Female (*n* = 12)
Overall	3 (25)	2 (16.7)	2 (16.7)	8 (66.7)
Abdominal pain	1 (8.3)	0	0	0
Constipation	0	0	0	1 (8.3)
Diarrhea	1 (8.3)	0	0	0
Hematochezia	0	0	0	1 (8.3)
Nausea	0	0	0	1 (8.3)
Dizziness	1 (8.3)	0	0	1 (8.3)
Headache	0	0	0	2 (16.7)
Presyncope	0	0	1 (8.3)	0
Influenza-like illness	1 (8.3)	0	0	0
Infusion site extravasation	0	0	1 (8.3)	0
Tenderness	0	0	0	1 (8.3)
Skin laceration	0	1 (8.3)	0	0
Thermal burn	0	0	0	1 (8.3)
Nasopharyngitis	0	1 (8.3)	0	0
Vulvovaginal infection	0	1 (8.3)	0	0
Hepatic enzyme increase	0	0	0	1 (8.3)
Back pain	0	0	0	1 (8.3)

## DISCUSSION

The present study was designed to determine the effect of age group or sex on the PK profile of isavuconazole after a single 200-mg oral dose in healthy nonelderly and elderly male and female subjects. Data were analyzed using PPK. PPK analysis was performed to see if there was any potential bias in sampling time points that might affect the exposures of subjects and also to determine the effects of age and sex on the PK of isavuconazole. The PPK modeling showed that the elderly female subjects had lower isavuconazole clearance values and higher exposure levels than the other groups in the study. However, the PK values determined for the elderly females differed by less than 1.5-fold from those seen with other groups, and the differences were not considered clinically meaningful. One possible explanation for higher exposures in elderly females might be the predominance of Asian subjects. Data analyzed from phase 3 studies showed that Asian subjects have higher exposures than Caucasian subjects (10). Since the primary aim of this analysis was to see the effects of sex and age on the PK of isavuconazole, race was not analyzed and added as a covariate. The findings of our present study support the findings from two large phase 3 SECURE and VITAL clinical trials, which showed that age group and sex did not affect the PK of isavuconazole (10, 12).

While there have been a number of studies on the PK and pharmacodynamics of other azoles (reviewed elsewhere [4, 14–18]), few have directly investigated the effect of age group or sex. One report showed that the mean *C*_max_ (maximum concentration of drug in serum) and AUC values for voriconazole were higher in young healthy females aged 18 to 45 years than in young healthy men, but no significant differences were observed between healthy elderly men and elderly women (≥65 years) in the same study (14). However, in contrast to our results determined for healthy nonelderly and elderly subjects, voriconazole plasma concentrations were higher in elderly subjects than in younger subjects (14). Another study showed that voriconazole exposure tended to increase with age and weight but that the increase was not clinically meaningful (19). Furthermore, in one study, age and sex were shown to have no clinically meaningful effects on the PK of posaconazole (20, 21). None of these studies of other azoles indicated that an adjustment of the dose was required on the basis of the age group or sex of the recipients.

Isavuconazole given as a single dose was safe and well tolerated in the elderly subjects as well as in the nonelderly subjects. TEAEs were reported more frequently in the elderly female subject group; however, all TEAEs reported were mild in intensity and no clinically relevant effects with respect to vital signs or physical examination findings were observed in this study. Although PPK analysis showed that elderly female subjects had lower CL values and higher levels of exposure than the other groups of subjects investigated, higher exposures in elderly females were not associated with significant toxicity, as measured in this study. Therefore, no dose adjustment of isavuconazole appears to be necessary based on either the age group or sex of individuals.

## MATERIALS AND METHODS

### Study.

This was a phase 1, open-label, parallel group, single-dose study to assess the PK of isavuconazole in healthy male and female adults (ClinicalTrials.gov registration no. NCT01657890). The study was conducted in accordance with the Declaration of Helsinki and the International Conference on Harmonisation Guidelines for Good Clinical Practice. An institutional review board at the study center approved the protocol and all amendments. All subjects provided written informed consent prior to enrollment.

### Subjects.

Subjects were eligible for the study if they were healthy nonelderly adults aged between 18 and 45 years or were healthy elderly adults aged ≥65 years or had a calculated creatinine clearance value that was within the age-appropriate normal range or that, if abnormal, was not clinically significantly abnormal. Additionally, the body weight and body mass index (BMI) of eligible subjects were required to be at least 45 kg and between 18 and 32 kg/m^2^, respectively, and the 12-lead electrocardiograph (ECG) test results were required to be normal at screening and on day –1 or, if abnormal, not clinically significantly abnormal, as determined by the investigator. Aspartate aminotransferase, alanine aminotransferase, and total bilirubin levels were to be within the normal reference range.

Exclusion criteria included nonelderly subjects with any clinically significant disease history of a pulmonary, gastrointestinal, cardiovascular, hepatic, respiratory, neurological, psychiatric, renal, genitourinary, endocrine, metabolic, dermatologic, immunological, hematological, inflammatory, or malignant condition(s) (excluding nonmelanoma skin cancer). Other exclusion criteria included the following: history of cardiac disorders; viral, bacterial, or fungal infection 7 days prior to baseline visit or vaccination 30 days prior to baseline visit; hepatitis B, hepatitis C, or HIV positivity; and/or a medical or surgical condition that might have interfered with the absorption, distribution, metabolism, or excretion of isavuconazole.

### Study design.

All subjects received a single dose of 372 mg of isavuconazonium sulfate, corresponding to 200 mg of isavuconazole, on day 1, after fasting for approximately 10 h prior to dosing. Subjects attended the study center from day –1 to day 4 and returned to the center for outpatient assessments on days 6, 8, 11, 13, and 15. Serial blood samples for isavuconazole PK were collected predose and at 0.5, 1, 1.5, 2, 3, 4, 6, 8, 10, 12, 16, 20, 24, 36, 48, 72, 120, 168, 228, 240, and 336 h postdose.

### Safety assessments.

The safety analysis set consisted of all participants who had one oral dose of isavuconazole. Safety assessments included an evaluation of the incidence, nature, and severity of adverse events, TEAEs, drug-related TEAEs, and ECG and vital sign measurements, including 12-lead ECG.

### Data analysis.

Noncompartmental analysis was performed on the PK data, which are not presented in this article. However, some of the parameters might not have been reliable due to insufficient sampling time in the elimination phase. Therefore, PPK was performed to estimate parameters with reliability. A total of 882 concentrations from 48 subjects were used for modeling. PPK modeling was performed using the concentration-time data in both elderly male and female subjects and nonelderly male and female subjects using nonlinear mixed-effects modeling with the software program NONMEM (version 7.2; GloboMax LLC, Hanover, MD). The first-order conditional estimation methods in NONMEM were employed for all model runs. Model selection was driven by data and based on various goodness-of-fit criteria, which included visual inspection of diagnostic scatter plots (observed concentration versus individual predicted concentration), successful convergence of the minimization routine with at least two significant digits in parameter estimates, precision of parameter estimates, and the minimum objective function value and the number of estimated parameters.

### Structural pharmacokinetic model.

A variety of linear compartmental models were explored to describe total isavuconazole concentration-time data. The base PPK model included two compartments, with either simple first-order absorption models or the Weibull absorption model. All random effects were treated as log-normally distributed. The ln-ln transformations of both the model and the data were used to stabilize the residual variance. The residual variance was finally modeled as additive in nature. The models were coded using NONMEM subroutines for prediction of PPK parameters. Initially, different age groups/sex groups (nonelderly males, elderly males, nonelderly females, and elderly females) were built directly into the base model by allowing each group to have their own unique population mean CL values since the aim was to determine if PK values differed based on age or sex.

### Pharmacokinetic model with covariates.

Following development of the base structural PPK model, a covariate analysis conducted to determine stepwise covariate modeling was performed in PSN (https://uupharmacometrics.github.io/PsN/) by the use of forward-inclusion and backward-elimination steps. In the forward-inclusion step, covariates (age, weight, BMI) were added to the model one at a time, using a *P* value of 0.01 for entry into the model. Univariate analysis of all specified covariate-parameter relationships was explored. The best covariate was added to the model, and the univariate step was repeated with the remaining covariates. This process was continued until no significant covariates were left to be added to the model. In the backward-elimination step, covariates were removed one at a time using a *P* value of 0.01 for retention in the model. The process was continued until all remaining covariates were statistically significant. The total AUC at steady state for individual subjects was calculated using the standard formula (AUC = *F* × dose/CL, where *F* is bioavailability and CL is clearance) based on the individual parameter estimates from the best covariate model.

### Statistical analysis of AUC.

To assess the effect of subject status as elderly females/nonelderly females versus males, an analysis of variance was conducted with log transformed AUC. The ratios of geometric means and the corresponding 90% confidence intervals were provided.

### Model validation.

For the best covariate model, population and individual PK parameters were estimated and the values representing the precision of the population model parameters (e.g., asymptotic standard errors or bootstrap 95% CIs) were generated. Nonparametric bootstrapping was used with 500 replications to provide validation of the model parameter estimated. The NPDE data were also plotted to evaluate the best model.

## Supplementary Material

Supplemental file 1
