# Supplementary material for: No Dose Adjustment for Isavuconazole Based on Age or Sex
Source: Antimicrob Agents Chemother. 2019 May 23;63(6):e02629-18. doi: 10.1128/AAC.02629-18 (PMC6535513; doi:10.1128/AAC.02629-18)
Supplement: Supplemental file 1 [file AAC.02629-18-s0001.pdf]

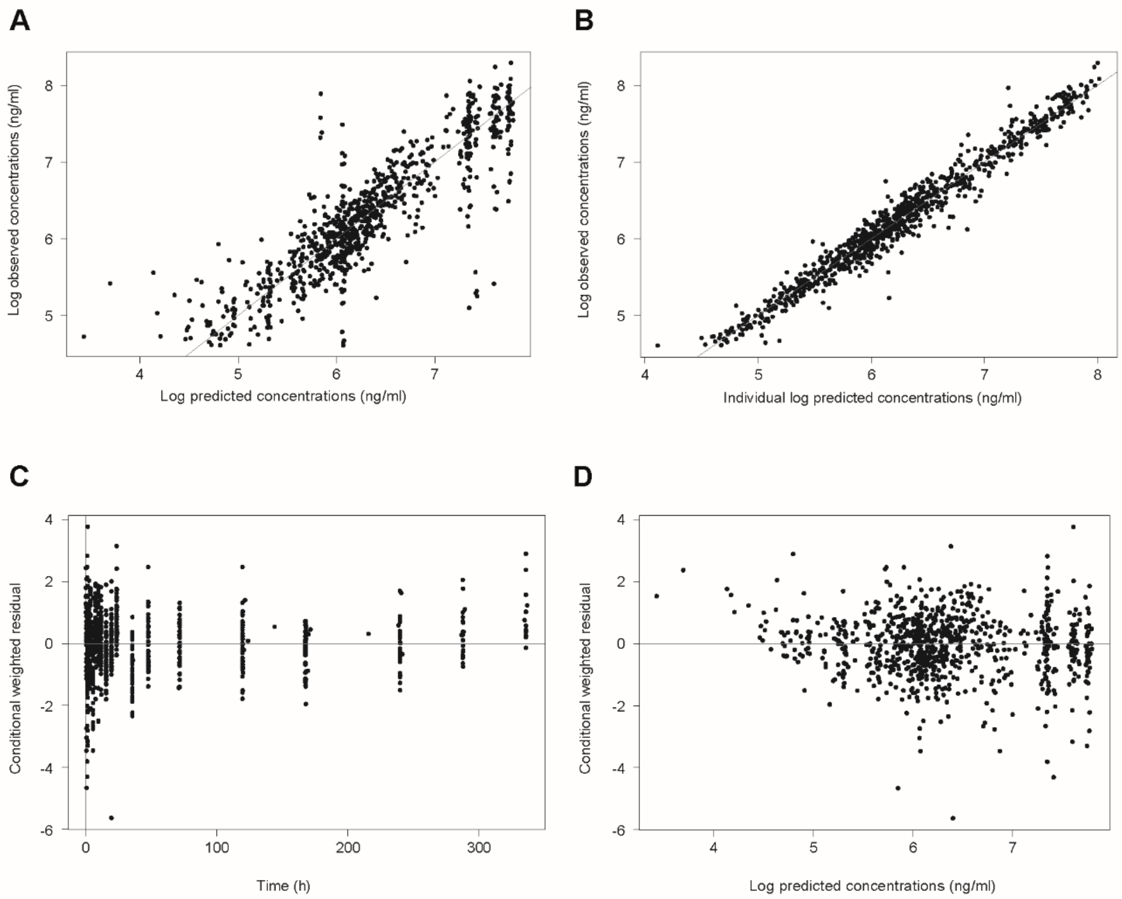

SUPPLEMENTARY FIG 1 Goodness of fit plots for the best population pharmacokinetic model. (A) Log of predicted concentrations versus log of observed concentrations. (B) Log of individual predicted concentrations versus log of observed concentrations. (C). Plot of conditional weighted residual versus time. (D) Plot of conditional weighted residual versus log of predicted concentrations.

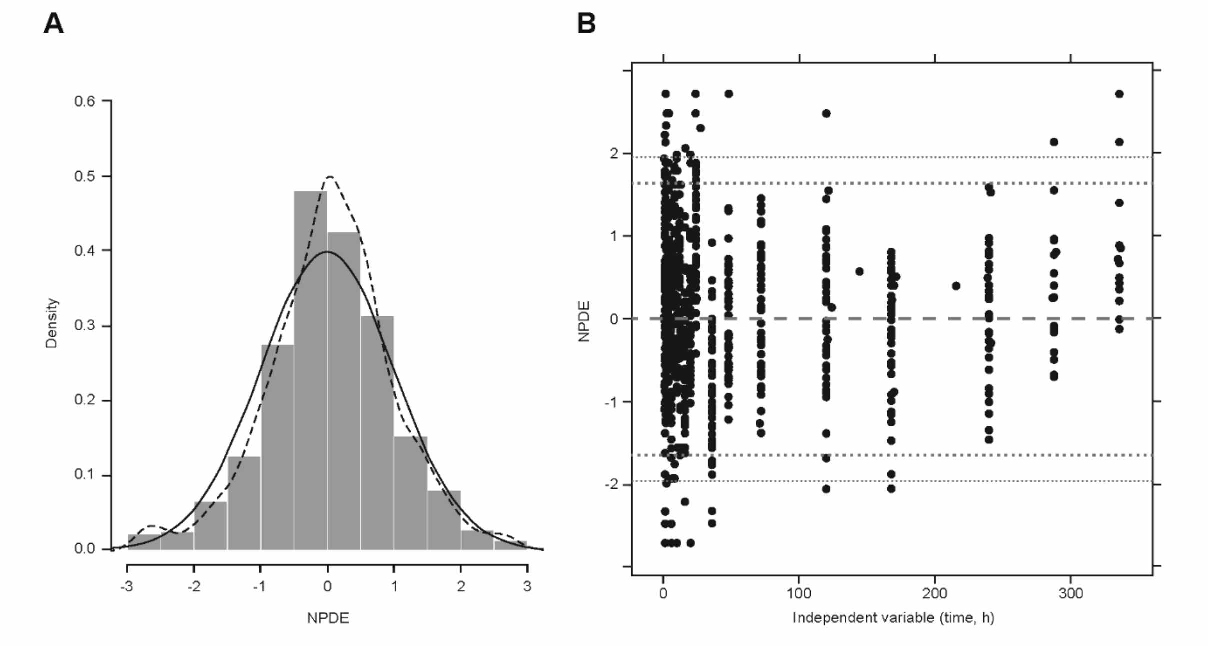

SUPPLEMENTARY FIG 2 Plots of normalized prediction distribution error (NPDE).

(A) Histogram of NPDE with the density of normal distribution and variance, N (0,1), with overlays of normalized distribution (solid line) and calculated distribution (dashed line). (B) Plot of NPDE versus time (dotted lines represent 90% and 95% prediction intervals).
